# Interfering growth of malignant melanoma with Ang2-siRNA

**DOI:** 10.1007/s11033-012-2189-4

**Published:** 2012-11-16

**Authors:** Biao Wang, Zhaoliang Liu, Mingfeng Zhang, Xiuying San, Yanding Zhang, Weiqiang Zhang, Meishui Wang

**Affiliations:** 1 Department of Plastic Surgery, First Affiliated Hospital of Fujian Medical University, Road Chazhong 20, Taijiang, Fuzhou, 350005 China; 2 College of Life Sciences, Fujian Normal University, Fuzhou, 350108 China

**Keywords:** *Ang2*, Lent virus, RNAi, Malignant melanoma

## Abstract

To investigate the intervention therapy effect on the growth of malignant melanoma, we have made an observation of expression levels of Ang2 in malignant melanoma cells, which was transduced by small interfering RNA (Ang2-siRNA) of Ang2 in vitro and in vivo. We successfully constructed Ang2-siRNA lent virus, and constructed nude mice model by transplanting malignant melanoma. Ang2-siRNA lent virus inhibited *Ang*2 mRNA of malignant melanoma in vitro and in vivo, and inhibited malignant melanoma tumor growth at the same time. Ang2-siRNA lent virus can interfere expression levels of Ang2 in malignant melanoma cells, inhibit tumor growth, and silent *Ang*2 gene expression, which may pave a new way for clinical gene therapy of malignant melanoma.

## Introduction

Angiogenesis is extremely important in multiply physiological and pathological processes [1]. When there are a large number of angiogenic cytokines, neovascular formation will be activated in vivo, which may cause tumorogenesis. The angiopoietin (Ang) family is a newly discovered group of cytokines with specific roles in regulation of vascular endothelial. Ang2 is a member of the family [2], which is one of the important initial factors in tumor angiogenesis and proliferation of endothelium [3], related with the density of tumor blood vessels, tumor stage and prognosis of survival closely [4]. Ang2 widely expresses in vascular system of human tumors, but very limitedly in normal tissues. Our previous experiments have been confirmed to inhibit the expression of *Ang2* gene can suppress angiogenesis [5], making it become an attractive candidate target in anti-angiogenesis in cancer therapy [6].

Malignant melanoma is a type of human tumor, formed from transformation of malignant melanocytes, with the characteristics of strong invasion and metastasis abilities, as well as poor prognosis. Its prognosis is particularly affected by low sensitivity of conventional treatments such as chemotherapy, radiotherapy and immunotherapy, mainly in immune and gene therapy in developed countries, besides invasion and metastasis abilities [7]. RNA interference (RNAi) is a gene silencing process mediated by small interfering RNA (siRNA) which is transferred from double-stranded RNA (dsRNA) [8]. siRNA guides RISC to specifically degradate the homologous mRNA through pairing the specific homologous fragment, leading to the inhibition of gene expression. Therefore, a small fragment of siRNA can induce efficient gene silencing [9], and thus it can make use of RNAi to block the target gene expression to achieve the purpose of diseases therapy.

Lentiviral vectors can infect non-dividing cells. The target gene can be integrated into the genome of infected cells, maintain long-term expression and cause limited immune response, therefore it has become an ideal gene transfer vector with widespread concentration because of its advantage. Lentiviral vector and can express stable siRNA against Ang2 long-term in tissues, whose gene shows high expression in malignant melanoma [10], so we can build of Ang2-siRNA lentivirus, and make use of its interference with the *Ang2* gene expression to inhibit tumor angiogenesis, vessel growth, and tumor proliferation, in order to provide a theoretical basis for clinical treatment of malignant melanoma.

### Methods

#### Restriction sites of the plasmid

In order to determine *Xba*I restriction enzyme sites are located at pre-constructed plasmid vector, plasmid pSilencer 1.0-U6-Ang2-siRNA and plasmid pNL-EGFP, we used plasmid map analysis by Gene Tool software.

#### Lent viral production and transduction

We recovered and purified the Ang2-siRNA fragments and pNL-EGFP fragments, which contained the U6 promoter, through being digested by *Xba*I, with electrophoresis, and the gel extraction kit, and dephosphorylated the recycling pNL-EGFP terminal at the same time. PNL-EGFP fragments and ang2-siRNA fragments were connected at a condition, of which molar concentration ratio is 10:1, products were transduced into DH5a cells, cultured at Amp + LB plates. Screening positive colonies to extract the recombinant plasmid, the extracted recombinant plasmid were digested by *Xba*I restriction enzyme, and identified by electrophoresis. After restriction enzyme digestion, and electrophoresis identification of the recombinant plasmids, correct recombinant plasmids were sequenced.

#### 293T cells were trans infected using the CaPO4 method, producing EGFP viral 293T

We take 10 plates of 10 cm of 70 % cell abundance 293T cells, adding 10 μl chloroquine of 25 mM in each plate, mixed gently, put back into the incubator for 1 h, and then changed the cultural media. We took X μl (100 μg) pNL-EGFP Y μl (70 μg) pHelper, Z μl (60 μg) pVSVG, and added into the 50 ml centrifuge tube and gently mixed, then added 450 μl 2.5 mol/l CaCl2, and TE supplement added to 6,250 μl, gently blowing mixed. 6,250 μl HEPES was added in drop wise, rapid mixing, put aside for 25 min. 10 plates of 293T cells was added with chloroquine, were replaced with new serum-free DMEM, adding 1,250 μl of the above mixture per plate, gently shaking, placed at the incubator for 10–12 h. We observed the expression levels of the reporter gene EGFP under an inverted fluorescence microscope, and then discarded the old medium, adding new serum-containing DMEM to terminate trans infection. After terminating trans infection at 12, 24, 48 h, it was observed by fluorescence microscopy, photographed, and saved, and then collected viral supernatant, it was then stored for further experiments.

Cells were plated at a density of 2 × 10^5^–4 × 10^5^ 293T cells per well in 6-well plates. When the cell abundance about 75 %, added the same amount of virus stock solution according to the gradient, respectively, diluted 105 virus solution. After incubated at 37 °C for 24 h, replaced DMEM medium and continued to foster. 24–48 h later, inverted fluorescence microscope was used to detect GFP expression levels to calculate the virus titer.

#### Infection of A375 cells by lent virus in vitro, and the detection of Ang2 mRNA level

Select multiplicity of infection (MOI) for 10,20,40,80 to infect A375 cells, blood cell counting plate to calculate the efficiency of infection, choose the appropriate multiplicity of infection. A375 cells in the logarithmic growth phase Digested by trypsin, and 2 × 10^4^/mL single cell suspension was made, cultured in 6 well culture plate. The experiment was divided into four groups: group A virus-infected cells, negative control group; group B was added the culture medium, blank control group; C and D adding different RNAi target virus-infected cell. According the MOI, adding the appropriate amount the virus for infection, after 2–3 days, inverted fluorescence microscope was used for observation, taking pictures, and was saved. Total cellular RNA were extracted from the A, B, C, and D group, trans scripted into cDNA reversely, Ang2 (183 bp) was amplified specifically, Ang2 forward (5′-GGGCATAATTGTGCTTGACTGG-3′), and reverse (5′-ATGGTCTTTAGAATTGG GTCACTGG-3′) primers, GADPH forward 5-GCACCGTCAAGGCTGAGAAC-3′), and reverse (5′-TGGTGAAGACGCCAGTGGA-3′) primers. GADPH was as an endogenous control for the implementation of the fluorescent quantitative RT-PCR to detect Ang2 mRNA expression. (7500 Real Time PCR System, Reps: 45–95 °C 5 s, 60 °C 34 s, 95 °C 15 s). Calculated RNAi inhibition of Ang2 expression efficiency (∆∆Ct = ∆Ct1 − ∆Ct2) by the formula N1/N2 = 2 − ∆∆Ct.

#### Xenografts in nude mice model

Collection of A375 cells in logarithmic growth phase, cells was digested by 0.25 % trypsin, and made into single cell suspension, adjust the cell density to approximately 5 × 10^7^/ml inoculated he 100 μl above A375 single cell suspension in nude mice right armpit subcutaneously. Nude living conditions and tumor formation were observed daily for the establishment of a nude mouse model.

#### Lent virus infection of A375 cells in vivo, and observation of tumor growth, Ang2 mRNA level detected

Measuring tumor length (L) and width (W) diameter every five days. By improving method to calculate the tumor volume of Tang et al. [12], Smoller et al. [13], according to the formula V = (L × W^2^) × 3.14/6 to calculate tumor volume. When the tumor grew to about 6 × 6 mm (22 days), nude mice were divided into blank control group, no-load group, experimental group, (*n* = 5). Nude mice of each group were treated with a multi-point injection of the tumor pNL-EGFP-of Ang2-siRNA lent virus solution (experimental group), pNL-EGFP lent virus solution (no-load group), PBS (blank group) 200 μl every other day, and intra-abdominal injection of 500 μl to for strengthening. Measuring and calculation of tumor volume, made tumor growth curve.

After 42 days of A375 cells inoculated, 4 % chloral hydrate intra peritoneal injection of nude mice for anesthesia, we then made incision of the tumor surface, and complete stripping of tumor. Total RNA from tumor tissue were extracted, and reversely transcribed into cDNA, was amplified by PCR instrument, amplification products were used for agarose gel electrophoresis image analysis; and the implementation of the efficiency of fluorescent quantitative RT-PCR to determine RNAi inhibition of Ang2 expression (7300 Real Time PCR System Reps: 40, 95 °C 5 s, 57.5 °C 1 min, 95 °C 30 s).

### Statistical analyses

SPSS11.5 was used for statistical analysis, independent samples *t* test, *P* < 0.05 was considered statistically significant.

## Results

### Identification of the vector

#### Plasmid pNL-EGFP and pSilencer1.0-U6-Ang2-siRNA digested by *Xba*I restriction enzyme

**Fig. 1:**
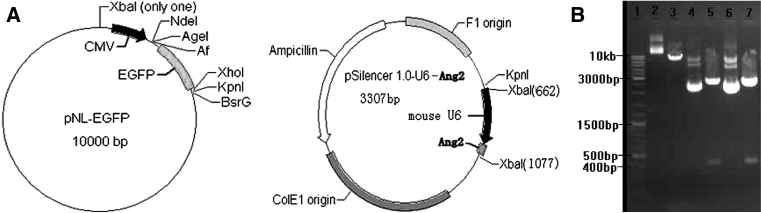
**a** Plasmid map of pNL-EGFP and pSilencer1.0-U6-Ang2. **b** Electrophoresis of plasmid digested by *Xba*I

**Fig. 2:**
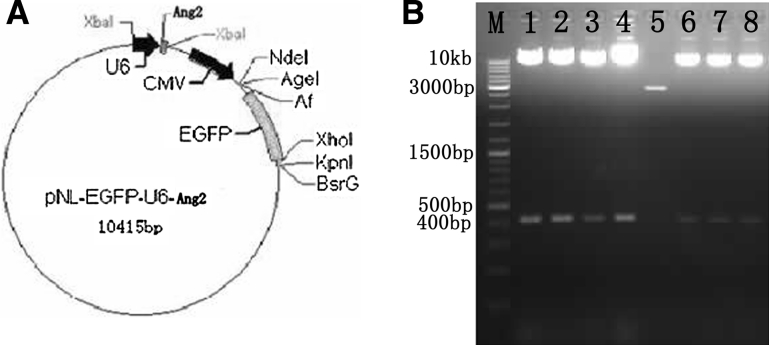
**a** Plasmid map of pNL-EGFP-U6-Ang2. **b** Plasmid pNL-EGFP-U6-Ang screened by *Xba*I

From the plasmid map of PNL-EGFP and pSilencer1.0-U6-Ang2-siRNA (Fig. 1a), there are *Xba*I restriction sites at both of the plasmid. There was only one *Xba*I restriction sites of pNL-EGFP, digested by *Xba*I to be a line molecular, showing a ladder by Electrophoresis (Fig. 2b, lane 3). There were 3 ladders if is not digested by electrophoresis. That is: super coiled circular plasmid, open-loop plasmid, open-loop with a gap in the plasmid (Fig. 2b, lane 2). There were two *Xba*I restriction sites at plasmid pSilencer1.0-U6-Ang2-siRNA. Plasmid pSilencer1.0-U6-Ang2-siRNA could be digested into fragments of a 3 and 400 bp. There were two ladders by electrophoresis (Fig. 2b, lanes 5, 7). If they were not been digested, which is a ring form, three ladders could be seen when electrophoresis (Fig. 2b, lanes 4, 6).

#### Identification of plasmid pNL-EGFP-U6-Ang2

Mixed the U6-Ang2 fragments from pSilencer1.0-U6-of Ang2-of siRNA plasmids with pNL-EGFP vectors (Fig. 1a) digested by the *Xba*I enzyme (Fig. 1a). Legating reaction of U6-Ang2 fragments and pNL-EGFP vectors at the molar ratio of 10:1, and screened positive clones were digested by *Xba*I, and identified by electrophoresis, if the connection was successful, U6-of Ang2 fragment would be cut (Fig. 2a).

**Fig. 3:**
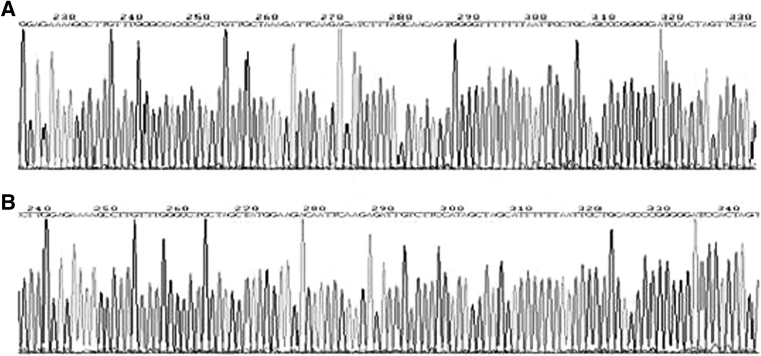
Identification sequencing of positive clones. **a** Identification sequencing of pNL-EGFP-U_∈_-Ang2-I. **b** Identification sequencing of pNL-EGFP-U_∈_-Ang2-II

Screened results: lane 1,2,3,4,6,7,8 may be the pNL-EGFP-U6-Ang2 (Fig. 2b). Positive clones were screened by restriction enzyme, electrophoresis, which was sequenced, the sequencing results showed that the connection was successful pNL-EGFP-U6-Ang2 plasmid strains were obtained (Fig. 3).

### Lent virus production

#### Co-trans infected 293T cells by plasmids pNL-EGFP, pVSVG and pHelper, obtained EGFP virus

293T cells were derived from human renal epithelial cell line 293 cells, which are triploid of human cell. Plasmid containing the replication origin sites and the promoter region of SV40 can be replicated in cells. When 293T cells were in the logarithmic growth phase, the pNL-EGFP, pVSVG, pHelper plasmids trans-ducted into the cells at the same time. If the green fluorescence observed under a fluorescence microscope after 12 h, it was indicated that transinfection was successful, and the media was changed immediately to terminate the process of transinfection reaction.

**Fig. 4:**
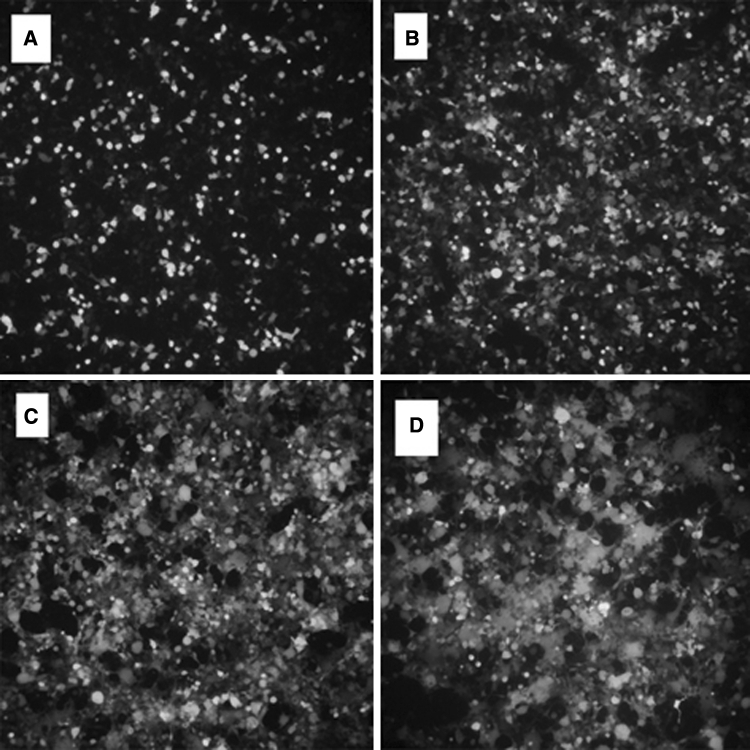
Process of the plasmids pNL-EGFP, pHelper and pVSVG co-transinfected in 293T cells (×10)

After the first 12 h of termination of the trans infection reaction, we observed bits and pieces of green fluorescence (Fig. 4a); barely into a piece of bright green fluorescence in the first 24 h (Fig. 4b); dazzling green fluorescence still being visible in the first 48 h, while some cells detached from the wall floating in the culture media (Fig. 4c); honeycomb-like fluorescence appeared after 60 h, the cell sheet was scattered in the culture media (Fig. 4d). That meant a large number of cellular lysates, which resulting in many EGFP virus.

#### Lent virus primary liquid titer

**Fig. 5:**
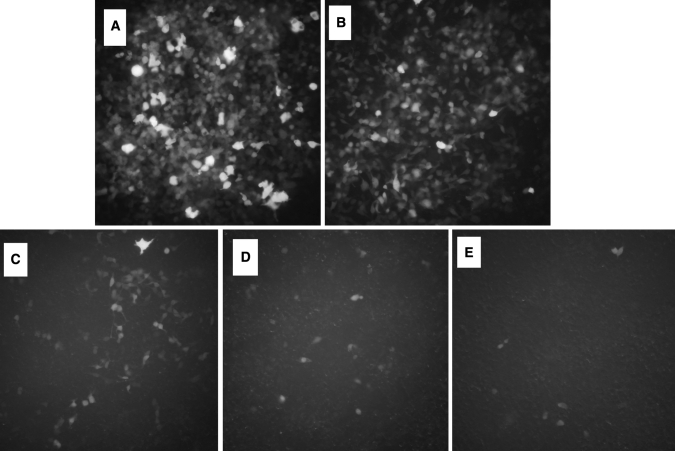
Lent virus infection at different concentrations in 293T cells (×10)

When 293T cells were in the logarithmic growth phase, the lent virus infected 293T cells with different concentrations and observed under fluorescence microscope. Infected cells with visible fluorescence could be seen in 5 pates infected cells, but the intensity of fluorescence was different: with the reduction of virus concentration, the brightness of the green fluorescence decreasing (Fig. 5). The titer value of lent virus primary solution = (42 × 104 × 10)/500 μl = 8.4 × 103/μl.

### Lent virus infection of A375 cells in vitro, and the detection of Ang2 mRNA level

#### Lent virus infection of A375 cells to determine the appropriate multiplicity of infection

**Fig. 6:**
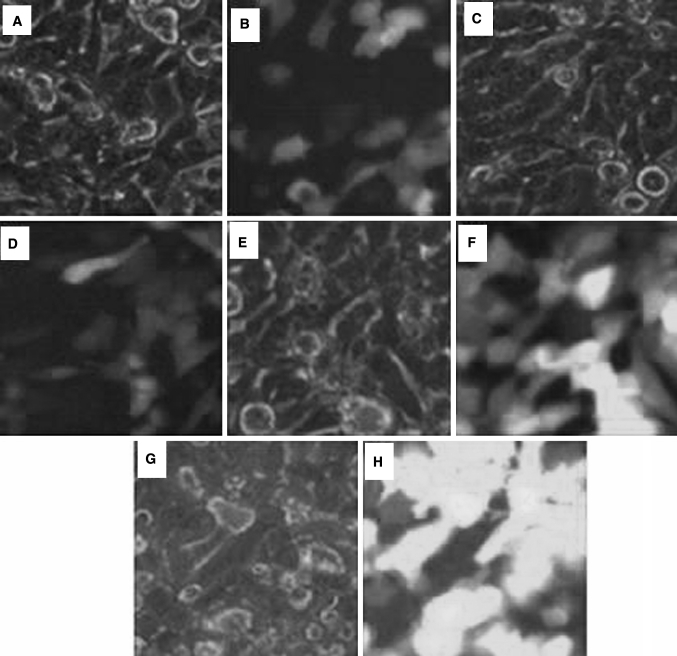
The situation of malignant melanoma cells in different multiplicity of infection conditions infection (×40)

When A375 cell growth state meet the requirements, we selected infected A375 cells, the multiplicity of infection of which was 10, 20, 40, 80, and observed under fluorescence microscope camera (Fig. 6), the higher of the multiplicity of infection, the brighter of the green fluorescence. The cells were digested into single cell suspension, counting with blood cell count plate (Table 1), the appropriate multiplicity of infection of 80 was used in the subsequent experiments.

**Table 1:** The efficiency of lent viral infection of malignant melanoma cells

MOI	Fluorescence cell count under normal light	Mercury light	Cell count under infection efficiency (%)
80	106	112	94.6
40	93	106	87.7
20	83	108	76.9
10	83	118	70.3

#### Ang2 mRNA level by real-time fluorescence quantitative RT-PCR

**Fig. 7:**
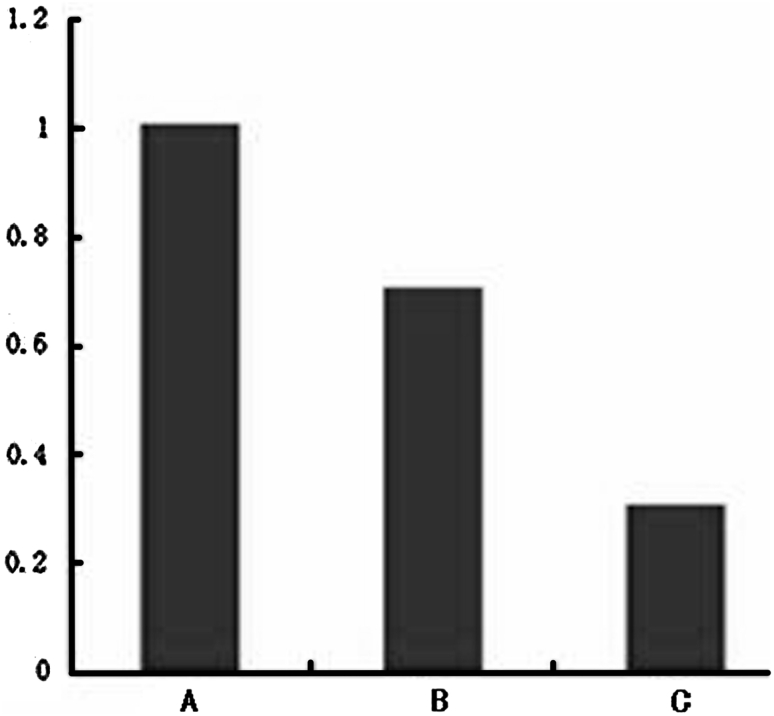
The efficiency of Ang2 expression suppressed by RNAi. *A* Control group, the relative expression levels of Ang2 was 100 %, *B* EGFP-Ang2-I lent virus infection, the relative expression levels Ang2 was 31.69 %, *C* EGFP-Ang2-II lent viral infection, the relative amount expression of Ang2 was 70.71 %

Total RNA of A375 cells were extracted from group A, B, C, D, and then reversely trans-scripted into cDNA, respectively. Drawing the same amount of cDNA template, real-time fluorescence quantitative RT-PCR was performed for Ang2 to obtain RNAi inhibition of Ang2 gene expression efficiency (Table 2, ∆Ct = Ang2 Ct—GADPH Ct; mean ∆∆Ct = mean ∆Ct1−mean ∆Ct2). This meant that EGFP-Ang2-I lentivirus suppressed the Ang2 gene expression by 68.31 % (Fig. 7), and the difference was statistically significant (*P* < 0.05); EGFP-Ang2-II lent virus suppression efficiency is low, the difference was not statistically significance (*P* > 0.05), also groups A and B inhibition efficiency was not statistically significant (*P* > 0.05). So Ang2-EGFP lent virus was used in the Subsequent animal experiments.

**Table 2:** The efficiency of RNAi inhibition of Ang2 mRNA expression level in the experimental group

Group (*n* = 3) inhibition rate (%)	Mean ∆Ct (${\bar{\text{X}}}$ ± S)	Mean ∆∆Ct	Expression
A (Ct)	18.802 ± 0.544	0	
B (Ct)	19.008 ± 1.109^a^	0.206	
C (Ct)	20.460 ± 0.553^b^	1.658	68.31
D (Ct)	19.302 ± 0.454^c^	0.5	29.29

Group B and A: ^a^ *P* > 0.05, Group C and A ^b^ *P* < 0.05, Group D and A ^c^ *P* > 0.05

### Lent virus interfere with the growth of transplanted tumors in nude mice in vivo

**Fig. 8:**
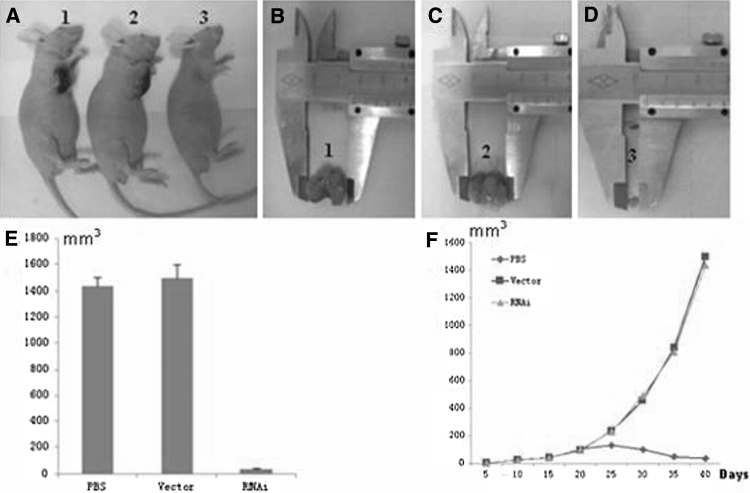
Differences in transplanted tumor of nude mice. **a** Subcutaneous tumor of nude mice at the end of experiment, group *1*, *2*, *3*, represent the blank group, the no-load group, the experimental group, and the nude mice subcutaneous tumor. **b**, **c** and **d**, for the measurements of subcutaneous tumor of blank group, no-load group, the experimental group, respectively. **e** Diagram of tumor volume, **f** tumor growth curve

We inoculated A375 cells subcutaneously in the right armpit to establish xenografts in nude mice. After an incubation period of 5–7 days, tumor could be visible at the inoculated parts of nude mice. After 22 days of inoculation of the A375 cells, nude mice tumors could grow to a size of approximately 6 × 6 mm. Intervention experiment was carried on, and then observation, measurement of subcutaneous tumor size of the blank group, the no-load group, and the experimental group (Fig. 8a–e). After inoculation of nude mice, measured and calculated of tumor volume at 5, 10, 15, 20, 25, 30, 35, and 40 days, and then drawn tumor growth curve (Fig. 8f).

There was no significant statistical difference between the blank group and no-load group (*P>*0.05). There was significant statistical difference between the experimental group and the blank group or no-load group (*P*<0.01).

### Ang2 mRNA levels of transplanted tumor in nude mice by implementation of fluorescent quantitative RT-PCR

Total RNA of were extracted from tumor tissues of the blank group, no-load group, and the experimental group, and then reversely transscripted into cDNA, respectively. Drawing the same amount of cDNA template, real-time fluorescence quantitative RT-PCR was performed for Ang2 to obtain RNAi inhibition of *Ang2* gene expression efficiency (Table 3).

**Table 3:** The efficiency of RNAi inhibition of Ang2 mRNA expression level in the experimental group

Group (*n* = 3)	Mean ∆Ct (${\bar{\text{X}}}$ ± S)	Mean ∆∆Ct	Experimental group (%)
No-load group (Ct)	8.865 ± 0.226	0	
Blank group (Ct)	8.988 ± 0.101^b^	0.123	8.17
Experimental group (Ct)	10.636 ± 0.385^a^	1.771	70.70

Comparison of the experimental group and the no-load group: ^b^ *P* < 0.05, Comparison of the blank group and the no-load group: ^a^ *P* > 0.05

**Fig. 9:**
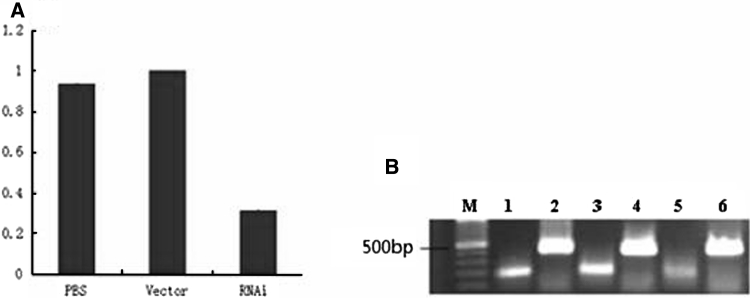
**a** The efficiency of RNAi inhibition of Ang2 mRNA expression level in the experimental group. **b** Expression level of Ang2 mRNA. *M* DNA Mark, *lane 1*, *3*, *5* was the blank group, no-load group, experimental group, and *lane* *2*, *4*, *6* was standard internal reference

Compared with no-load group, Ang2 mRNA expression level of the experimental group was decreased by 70.7 % (Fig. 9a), and the difference was statistically significant (*P* < 0.05); while *Ang2* gene mRNA expression level, compared with no-load group, blank group decreased by 8.17 %, no statistical significance (*P* > 0.05). Real time quantitative RT-PCR products were separated by gel electrophoresis and then pictured the result with a digital camera (Fig. 9b).

## Discussion

Our study was focused on the expression of *Ang2* gene and how it affected the growth of malignant melanoma via tumor angiogenesis and vessel growth. Ang2 is a member of angiopoietin family, secreted from the activated vascular endothelial and playing a role in promoting differentiation and activation of endothelial by autocrine pathway [14, 15], which achieves these biological effect depending on exogenous endothelial growth factor [16]. Its main function is competitively binding with Ang receptor Tie2, inhibiting the function of Ang1, including maintaining vascular blood perfusion, vascular series, maturity and structural stability. Ang2 can also enhance the sensitivity of endothelial to vascular endothelial growth factor, so that it promote vascular budding, growth and the formation of unstable vascular. Matsunaga et al. [17], Saito et al. [18] and other studies have reported Ang2 promoting vessel growth must rely on the presence of vascular endothelial growth factor. When lack of vascular endothelial growth factor, Ang2 inhibits role of Ang1, leading to vessels diminishing. Ang2 expression level in tumor tissue and circulating serum were significantly higher than that in normal tissue surrounding tumor or normal individuals. Helfrich et al. [19] found that Ang2 expression level was relative with the development process of metastatic malignant melanoma, and showed an important impact on the ability of tumor invasion and metastasis. While the majority of experiments show that the growth and metastasis of solid tumors depend on tumor angiogenesis [20], suppression of gene expression of Ang2, will affect tumor angiogenesis, vessel growth, moreover, regulate tumor growth.

Angiogenesis means formation of new vessels via budding pathway based on primary vessels. There are two main stages in proliferation and growth of solid tumors, non-vascular stage and vascular stage. It has not been reported that tumor own metastatic ability in non-vascular stage. Neo vessels in the tumor bring rich blood supply and nutrition from non-vascular stage to vascular stage. Because of poor stability of the neo vascular structures and the weak vascular wall, tumor cell s invade surrounding normal organization crossing vascular wall, or enter the blood circulation of long-distance systemic metastases. The mechanism of angiogenesis is the disturbance of the tumor growth microenvironment, the imbalance of pro-angiogenic and anti- angiogenic factors [22], over-expression of pro-angiogenic factors, and tumor angiogenesis. More severe, Maniotis et al. [23] found that subcutaneous melanoma had the vascular generation capacity independent on the budding of endothelial cells to form vascular, and also conduct blood and tissue fluid by duct which mimic vessel, deformed by its own cells and extracellular matrix, remodeling the tumor microenvironment.

According to the known sequence of mRNA of Ang2 as a target, we successfully constructed recombinant plasmid of pSilencer 1.0-U6-Ang2-siRNA. HUVECs were transfected by liposome-mediated transfection, testing expression level of Ang2 protein and mRNA, which was dominantly inhibited. Transfected HUVECs were cultured in three-dimensional culture model in vitro, and the number and length of vessel-like structures were significantly reduced, which denoted that angiogenesis was inhibited dominantly [5, 11]. All of the study illustrated that Ang2 could be a research object to study the anti-angiogenesis of tumor.

On the basis of previous study, we successfully constructed lent virus expression vector of Ang2-siRNA by application of transgenic technology and RNAi technology, which inhibited *Ang2* gene expression in malignant melanoma cells steadily. The primary virus liquid was used in the experiment, and the titer of the virus is relatively low, so we should select the appropriate multiplicity of infection for later experiment. When the efficiency of virus infection of malignant melanoma cells is 94.6 %, the multiplicity of infection is 80, so multiplicity of infection of 80 was used for infection test. The *Ang2* gene expression levels of malignant melanoma cell attenuated dominantly, causing inhibition of angiogenesis, which is an important progress of tumor genesis, so the inhibition of tumor growth, which provided a new method of therapy in clinic.
